# Protocol for Usability Testing and Validation of the ISO Draft International Standard 19223 for Lung Ventilators

**DOI:** 10.2196/resprot.7298

**Published:** 2017-09-08

**Authors:** Dev Minotra, Steven L Dain, Catherine M Burns

**Affiliations:** ^1^ Department of Systems Design Engineering University of Waterloo Waterloo, ON Canada; ^2^ Department of Electrical and Computer Engineering University of Waterloo Waterloo, ON Canada; ^3^ Woodstock Hospital London, ON Canada

**Keywords:** usability, terminology, standardization, lung ventilators

## Abstract

**Background:**

Clinicians, such as respiratory therapists and physicians, are often required to set up pieces of medical equipment that use inconsistent terminology. Current lung ventilator terminology that is used by different manufacturers contributes to the risk of usage errors, and in turn the risk of ventilator-associated lung injuries and other conditions. Human factors and communication issues are often associated with ventilator-related sentinel events, and inconsistent ventilator terminology compounds these issues. This paper describes our proposed protocol, which will be implemented at the University of Waterloo, Canada when this project is externally funded.

**Objective:**

We propose to determine whether a standardized vocabulary improves the ease of use, safety, and utility as it relates to the usability of medical devices, compared to legacy medical devices from multiple manufacturers, which use different terms.

**Methods:**

We hypothesize that usage errors by clinicians will be lower when standardization is consistently applied by all manufacturers. The proposed study will experimentally examine the impact of standardized nomenclature on performance declines in the use of an unfamiliar ventilator product in clinically relevant scenarios. Participants will be respiratory therapy practitioners and trainees, and we propose studying approximately 60 participants.

**Results:**

The work reported here is in the proposal phase. Once the protocol is implemented, we will report the results in a follow-up paper.

**Conclusions:**

The proposed study will help us better understand the effects of standardization on medical device usability. The study will also help identify any terms in the International Organization for Standardization (ISO) Draft International Standard (DIS) 19223 that may be associated with recurrent errors. Amendments to the standard will be proposed if recurrent errors are identified. This report contributes a protocol that can be used to assess the effect of standardization in any given domain that involves equipment, multiple manufacturers, inconsistent vocabulary, symbology, audio tones, or patterns in interface navigation. Second, the protocol can be used to experimentally evaluate the ISO DIS 19223 for its effectiveness, as researchers around the world may wish to conduct such tests and compare results.

## Introduction

Lung ventilators are frequently used in health care, and over 300,000 patients are ventilated in the United States every year [1]. However, the use of lung ventilators is associated with a number of complications and usage errors. While usage errors by clinicians can result in inadequate ventilation, overventilation, barotrauma, or patient-ventilator asynchrony, they can also worsen complications generally associated with ventilators, including ventilator-associated pneumonia, sepsis, psychological distress, acute respiratory distress syndrome, and pulmonary edema (all of which can increase the risk of patient disability and death) [1]. Moreover, medical device manufacturers use inconsistent nomenclature on user interfaces of lung ventilators. For example, the term *continuous mandatory ventilation* (CMV) can have different meanings on different ventilator models [2]. Similarly, a mode for volume-targeted pressure-controlled ventilation has five different names on different ventilator products (see Table 1, first row). While several such inconsistencies in terminology between different ventilator models exist (Table 1), there has also been considerable debate on the correctness of some of the terms used by manufacturers, and the extent to which terms can be intuitively interpreted by clinician users [3]. The discrepant nature of ventilator terminology is a factor underlying increased training costs and human errors, and is an impediment to communication between clinicians, electronic health records, and ventilators. Henzler [4] suggests that partial ventilatory support modes are ill defined, and studies conducted on these modes are difficult to interpret or compare, which necessitates new and precise definitions and taxonomies for ventilation modes. Human factors and communication issues are the two most frequent root causes underlying ventilator-related sentinel events that occurred between 2004 and 2015, which were reported to The Joint Commission [5]. According to The Joint Commission, a sentinel event is an event that results in patient mortality, permanent harm, or severe harm of temporary nature requiring intervention to sustain life; additionally, a sentinel event is not primarily related to the affected patient’s illness or underlying condition [5]. The discrepant nomenclature of existing lung ventilators is an issue that is inseparable from human factors, communication, and training.

The main objective of our study is to determine the ease of use, safety, and utility of standardized vocabulary as it relates to the usability of medical devices, compared to legacy medical devices from different manufacturers. We will focus specifically on lung ventilators and the “ISO Draft International Standard 19223 – Lung Ventilators and related equipment – Vocabulary and Semantics” [6]. An International Organization for Standardization (ISO) subcommittee has been working to standardize vocabulary for lung ventilators since 2006; the ISO Draft International Standard (DIS) 19223 is under development, and it may soon become an ISO standard. Our study will evaluate terms defined in the ISO DIS 19223 in the context of their use with lung ventilator user interfaces. The proposed research protocol aims to assess whether terms defined in the ISO DIS 19223 improve the usability of lung ventilators.

Little work has been done to evaluate the impact of a standardized terminology on the usability of medical devices, especially on transitions across heterogeneous devices from different manufacturers. Bakhshi-Raiez et al [7] tested the usability of a clinical information system that incorporated the Systematized Nomenclature of Medicine - Clinical Terms (SNOMED CT) in the registration of reasons for admissions into intensive care. The protocol involved a three-month on-site implementation of SNOMED CT, usability evaluations before and after the implementation, and 16 intensive care unit (ICU) physicians as participants [7]. However, the protocol did not involve comparing multiple heterogeneous systems or comparisons of two or more nomenclature systems [7]. Juvé-Udina [8] reported a usability evaluation of the Architecture, Interface, Terminology, Information, Nursing, and Knowledge (ATIC) terminology for the documentation of nursing diagnoses. The study involved a longitudinal design involving two hospitals and electronic records incorporating the ATIC terminology [8]. A limitation of this protocol is that it mainly focuses on the frequency and completeness of a terminology rather than efficiency, accuracy, or error rate. The Juvé-Udina protocol is not applicable in the evaluation of a nomenclature system intended to be used across heterogeneous devices [8]. Rosenbloom et al [9] proposed a model to evaluate clinical terminology used in the interaction between humans and structured clinical data. The model prescribes several terminology attributes (eg, concept coverage, term accuracy, term expressivity) and usability factors, including correctness, completeness, efficiency, and user satisfaction [9]. However, the model focuses on the evaluation of medical terminology rather than user interface(s) incorporating a given terminology, and the model does not prescribe a protocol for comparing several systems that incorporate alternative terminologies [9]. Morita et al [10] reported a study comparing the safety and user experience of four ventilator models; although their experimental protocol is informative for usability studies involving ventilators, their design is not concerned with nomenclature standardization. Therefore, our protocol to evaluate the usability of a standardized nomenclature applicable across user interfaces of several heterogeneous devices would be a contribution, and the protocol would be applicable in other terminology-related usability studies.

Usability is associated with task performance, which is in turn associated with risk of complications and ventilator-associated lung injuries. Our proposed study will experimentally examine differences in task performance (ie, human error rate, task completion times) and error type (emerging from the use of a standardized versus nonstandardized nomenclature) on lung ventilator user interfaces in clinically relevant scenarios. Our research question and hypothesis are stated as follows: *If medical device manufacturers consistently incorporate a standardized nomenclature, will there be fewer usage errors committed by clinicians operating medical devices unfamiliar to them after some training on the standardized nomenclature? And: Usage errors with unfamiliar medical devices will be lower when mode naming standardization is consistently applied on medical devices produced by all manufacturers.*

**Table 1:** Translation of terms between vocabularies.

Description	Type	Term in ISO DIS 19223	Term in PB-840	GE Engstrom
Mode in which pressure is adjusted from inflation to inflation, and a set target volume is delivered	Mode	Volume-targeted pressure control (vtPC)	Volume Control Plus (VC+)	Pressure Control Ventilation - Volume Guaranteed (PCV-VG)
Sleep apnea breathing therapy mode	Mode	Continuous positive airway pressure (CPAP)	Spontaneous (SPONT)	Continuous Positive Airway Pressure/Pressure Support Ventilation (CPAP/PSV)
Mode in which two pressure levels are set for spontaneous breathing	Mode	Bi-level positive airway pressure (also bi-level PAP or BPAP)	BiLevel	BiLevel
Ventilation-pattern in which a selected inflation-type (which is the primary inflation) is initiated at a set rate. Patient-trigger events may lead to additional primary inflations beyond the set rate	Mode class	Assist/Control Ventilation	Assist/control mode	“Assist control” mode is available through Volume Controlled Ventilation (VCV), Pressure Controlled Ventilation (PCV), Pressure Controlled Ventilation - Volume Guaranteed (PCV-VG) modes only
Ventilation-pattern in which a selected inflation-type (which is the primary inflation) is initiated at a set rate; patient trigger events cause support inflations in which spontaneous breathing may occur; primary inflations are synchronized with any spontaneous breathing through “synchronization windows”	Mode class	Synchronized intermittent mandatory ventilation (SIMV)	SIMV, “mandatory breaths” can be volume or pressure-based	Several modes on - Synchronized Intermittent Mandatory Ventilation (SIMV) are present in the Engrstrom; SIMV Pressure Controlled (SIMV-PC), SIMV Volume Controlled (SIMV-VC), and SIMV Pressure Controlled, Volume Guaranteed (SIMV-PCVG). SIMV-VC, SIMV-PC, and SIMV-PCVG use a “trigger window” which is different from “synchronization windows” mentioned in the ISO DIS 19223
Baseline airway-pressure (BAP) or pressure level set above ambient pressure at which unassisted breathing may occur, and/or inflations may be superimposed	Setting	Baseline airway-pressure (BAP)	Positive end-expiratory pressure (PEEP)	PEEP
Higher pressure level in the Bi-Level Mode	Setting	BAP_H_	PEEP_H_	Phigh
Lower pressure level in the Bi-Level Mode	Setting	BAP	PEEP_L_	Plow
High PEEP time or Inspiratory Time	Setting	BAP_H_ Time or t_H_	T_H_	Thigh
Low PEEP time or Expiratory Time	Setting	BAP Time or t_L_	T_L_	Tlow
Setting for duration of inspiratory phase	Setting	Inspiratory Time or t_I_	T_I_	Tinsp
Setting or measured quantity for airway pressure in an inspiratory or inflation phase	Setting, measured quantity	Inspiratory Pressure	P_I_	Pinsp
Tidal Volume	Setting	V_T_	V_T_	Tidal Volume or TV

To test this hypothesis, the proposed experimental study will compare performance declines resulting from the use of an unfamiliar ventilator model. These declines will be compared between two groups: a group of clinical participants provided with legacy ventilator models, and a group of clinical participants provided with ventilator models that are modified to include standardized nomenclature. The experiment will consider user interfaces on two types of ventilator models: one familiar to clinical participants and one unfamiliar to clinical participants. Multiple manufacturers provide lung ventilators, so clinicians (eg, respiratory therapists) within a given geographic region become trained and familiarized only with a subset of the lung ventilators that the global market is capable of providing. However, clinicians may encounter unfamiliar lung ventilators on an occasional basis. For each ventilator model’s user interface, a variant will be developed in which the original layout and navigational structure will be retained, while replacing its nomenclature with the ISO DIS 19223.

The familiar ventilator model will be the Puritan Bennett 840 (PB-840), and the unfamiliar model will be the General Electric (GE) Engstrom Carestation (referred to in this paper as *Engstrom*). The two ventilator models differ in the extent to which respiratory therapy trainees and practitioners are familiar with their interfaces and nomenclature systems. The comparison will provide insights on benefits or difficulties with the use of the ISO DIS 19223 on ventilator user interfaces. A related objective of our proposed study is to identify any terms in the ISO DIS 19223 associated with a high rate of human error. Such terms, if any, should be used with caution on instruction manuals and interfaces.

### Relevance to Patient Care

Usability of medical devices is of direct relevance to patient care. It is common for ventilators from several different manufacturers (with different terminology) to be used in hospital departments or different hospital units within a hospital system; this leads to increased risk in patient care [11]. Patients being mechanically ventilated are transported between different health care settings that include homes, emergency departments, long-term care facilities, and ICUs. These movements require clinicians to be able to switch between different nomenclature systems in life-or-death situations; during transitions of care, inconsistent terminology has resulted in clinicians losing valuable time in handoffs that involve translating ventilator use instructions [2]. Clinicians are often required to use ventilators unfamiliar to them, and inconsistent nomenclature contributes to the risk of ventilator-associated lung injury. There is a high risk of mortality in patients being mechanically ventilated who have an acute lung injury: the mortality is 24% for patients 15 to 19 years of age and 60% for patients 85 years or older [1]. There are many causes for ventilator-related sentinel events; however, human factors and communication issues were the most common factors underlying such events that occurred between 2004 and 2015, according to The Joint Commission [5].

Human factors and communication issues are inseparable from the issue of nomenclature inconsistency. The development of an ISO standard for lung ventilator nomenclature is a positive step towards mitigating patient safety risk with the use of lung ventilators. Our research protocol (and eventual study to evaluate the ISO DIS 19223) will help us better understand the potential benefits and barriers, if any, in the incorporation of the ISO DIS 19223 in lung ventilators.

The results of our proposed study will be valuable in the adoption of the ISO DIS 19223 in lung ventilators and in training programs and manuals. When the ISO DIS 19223 becomes widely used, insights from our proposed study will be useful to interface designers of lung ventilators. The proposed study plans to recruit experienced respiratory therapists, so any frequently recurring error(s) associated with specific ISO DIS 19223 term(s) may be of interest in clinician education, in manuals, and in the design of training materials incorporating the ISO DIS 19223. This proposed study will also provide insights about relationships between operator mental models, device nomenclature, and operator error types, which would be a useful human factor contribution applicable to other types of medical devices and instruments.

### Present State of Knowledge and Practice

Lung ventilator models differ between manufacturers in terms of nomenclature. Differences are seen in definitions of various ventilation modes and other terminology, which affects training costs and human error in health care settings. The complexity of lung ventilators in use is a factor underlying patient complications and ventilator-associated lung injury. As an example of an adverse event, a 28-year-old in a neurological ICU was having difficulties with his/her ventilator; a respiratory therapist from a cardiothoracic ICU attending to this patient decided to change settings on the ventilator to improve the patient’s oxygen saturation [2]. This action resulted in barotrauma and deteriorated the condition of the patient; later, the respiratory therapist stated that he was not familiar with the ventilator and that the one (familiar to him) in his unit was different [2]. Adverse events like this necessitate the adoption of a standardized nomenclature system and require clinicians to become familiarized with the standardized terminology. As the current state of ventilator nomenclature is in disorder, human error associated with issues in communication, human factors, usability, and training cannot be mitigated unless a standardized terminology is implemented across manufacturers. The implementation of the standardized terminology would significantly benefit from a study focusing on assessing its usability and on the identification of any potential terms associated with conflict, frequent error, or misinterpretation.

As an example of conflict resulting from current inconsistencies in terminology, the term “breath” sometimes refers to an inflation performed by a lung ventilator, leading to ambiguity with the use of manuals and descriptions [12]; for disambiguation, it is argued that ventilators do not “breathe”, and the term “inflation” be used for referring to work done by a ventilator filling air in a patient’s lungs [12]. In 2010, there were at least 34 ventilator models using 174 unique terms for ventilation modes [13]. Additionally, terms for settings and modes are often abbreviated, which makes it more difficult for clinicians to familiarize themselves with an unknown ventilator model [14]. This example only provides an estimate of the extent of complexity and disorder with the current ventilator vocabularies in use.

Chatburn [11] stressed the need for a standardized vocabulary and taxonomy in lung ventilators to develop a better understanding about their scope and capabilities; the lack of a standardized vocabulary or nomenclature has jeopardized delivery of care, clinician training, and ventilator sales [11]. Chatburn provides a ventilator mode taxonomy that has been reportedly published for 15 years [11]. However, Chatburn’s terminology has not kept pace with changes in technology and the emergence of new modes and settings in lung ventilators. Therefore, an ISO subcommittee was formed to create an ISO standard.

In a 2014 Association for the Advancement of Medical Instrumentation/Food and Drug Administration summit on ventilator technology, it was noted that gaps exist in current clinical training for ventilator use and in the current state of ventilator terminology [2]. The lack of a, “simple, common, usable ventilator taxonomy including nomenclature” was reiterated by Dr. Steven Dain [2]. A number of related barriers in communication between ventilators and ancillary systems, and an inconsistent understanding of ventilator terminology, were highlighted [2]. A summit presenter noted that in the St. Louis region, five ventilator models in use had five different terms that referred to the same mode for *volume-targeted pressure controlled ventilation*. User interface issues (eg, interface layouts and navigation) were cited in addition to issues in model use competency, and in the setup of ventilators and alarm conditions [2]. It was noted that several barriers exist in training clinicians on lung ventilator models; this includes inadequate incentives to attend training programs conducted by manufacturers [2].

Advances in the standardization of lung ventilator nomenclature will have the most impact if user interfaces on lung ventilators and manuals adopt the standardized nomenclature. There has been no research done to examine or compare the effectiveness of two or more nomenclature systems in lung ventilators. The effectiveness of user interfaces is captured in the construct of usability. Therefore, usability is being incorporated in the proposed study to understand the potential impact that a standardized terminology could have. Usability is a multi-faceted construct that takes into consideration efficiency, errors, memorability, learnability, and satisfaction [15]. We believe that all components of usability are relevant in terms of evaluating the effects of adopting a standardized nomenclature.

In summary, our paper reports an experimental protocol to evaluate a standardized nomenclature system for a medical device, and the evaluation will determine the extent to which the standardized nomenclature facilitates the work of clinicians in situations in which the clinician would need to operate an unfamiliar medical device. The evaluation will take clinician error, performance, and usability into consideration. The medical device we will focus on is the lung ventilator, and the standardized nomenclature is the ISO DIS 19223.

## Methods

To test our hypothesis pertaining to nomenclature in medical equipment, the proposed research protocol focuses on lung ventilators while planning to involve respiratory therapy practitioners and trainees. The study will involve at least two lung ventilator models, one of which would be less popular in practice in the region of study, while the other would have a high level of familiarity due to common practice among clinical practitioners and trainees in the region of study. Mockups of interfaces on these ventilator models will be developed. Additionally, for each ventilator model being considered in the study, a variant incorporating the terminology in the ISO DIS 19223 will be developed. This protocol will involve the following tasks: (1) *development of materials - training, mockups, and scenarios*, (2) *vetting and refinement of materials*, (3) *recruitment of clinical participants*, (4) *experiment*, and (5) *analysis*.

### Task 1: Development of Materials - Training, Mockups, and Scenarios

In Task 1, we will develop mockups of the *familiar* PB-840 ventilator and the *unfamiliar* GE Engstrom Carestation. The contrast in familiarity is expected in the targeted participant sample in the region of study. Based on our interaction with subject matter experts, one of these products is less commonly used in the market within the region of study. A ventilator mockup is a scaled-down simulator that will consist of a series of screens on the ventilator that allows input-driven transitions between screens. Each screen will be similar to that of the corresponding ventilator. The Department of Systems Design Engineering at the University of Waterloo has a PB-840 ventilator and a GE Engstrom Carestation. These ventilators were provided for educational and research use by Puritan Bennett and GE.

There will be a total of four mockups (PB-840, PB-840-ISO, Engstrom, and Engstrom-ISO), as a variant for each model will be developed using of the ISO DIS 19223 nomenclature. In Phase 1, we will also prepare 10 clinically relevant scenarios, of which seven will be routine scenarios and three will be nonroutine critical scenarios. Each scenario will require the participant to specify or change modes and/or settings on a ventilator mockup. The training materials will be designed to familiarize participants with the ventilator mockups. Training materials for the PB-840, the GE Engstrom Carestation, and their ISO-standard variants will be prepared. Definitions of terms used on the ventilators will not be provided in training.

### Task 2: Vetting and Refinement of Materials

The objective of this task is to get feedback on experimental materials (ie, clinical scenarios, mockups) and the experimental protocol. This feedback will be elicited from experienced respiratory therapists and we will use the feedback to refine the experimental materials and protocol. The task will mainly involve a pilot run of the main experiment with think-alouds and unstructured interviews. We will recruit approximately 12 experienced respiratory therapists [10] and instructors from the respiratory therapy program at Conestoga College. The pilot trial and feedback will be used to make modifications in the materials. We will request that participants pay attention to the following areas: (1) comprehensibility of scenario descriptions and discovery of any ambiguities; (2) identification of scenarios that are too difficult to solve by experienced respiratory therapists (we will remove or modify any scenarios for which accurate answers cannot be obtained for most respiratory therapists in the control condition); (3) do the mockups appropriately represent the user interfaces of the PB-840 and the GE Engstrom Carestation?; and (4) is it easy to follow the training, and what would be the appropriate training time for familiarization with the ISO DIS 19223 or that of the unfamiliar ventilator model?

### Task 3: Recruitment of Clinical Participants

**Figure 1:**
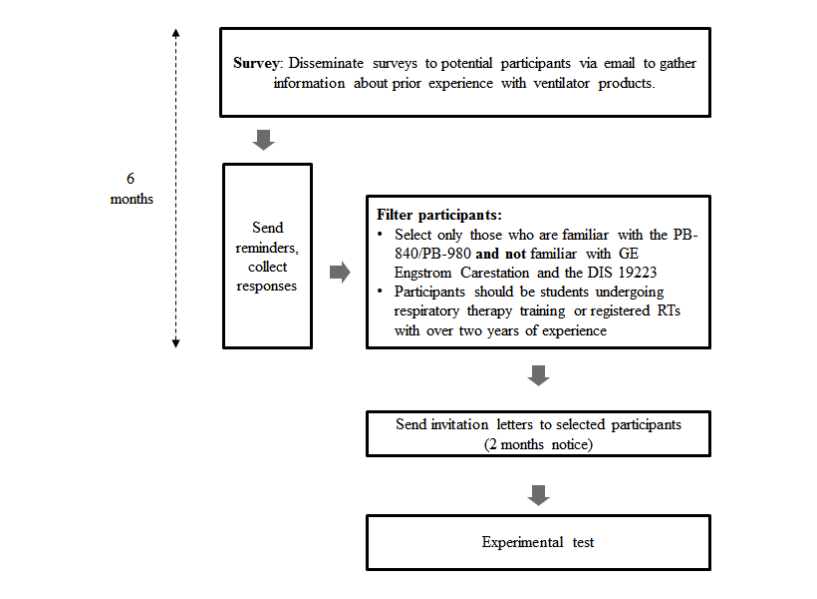
Planned timeline for recruitment of clinical participants. PB-840: Puritan Bennett 840 ventilator; GE: General Electric; DIS: Draft International Standard; RT: respiratory therapist.

We plan to recruit 60 participants with training and/or clinical experience with ventilators. A power analysis was conducted with G*Power. Considering a medium effect size (effect size f of .25), alpha of .05, power of .80, and the assumption of a moderate correlation (r=.5) between performance measures across ventilator types, the required sample size is estimated to be 48 participants. However, participant data may need to be excluded due to performance considerations or technical issues. Therefore, a target participant pool of 60 would be appropriate for this study. This sample will consist of 30 students undergoing training in respiratory therapy and 30 registered respiratory therapists who have been in practice for over two years. This timeframe is equal to (or higher than) the experience level of *Therapist II* in the American Association for Respiratory Care’s career ladder model; *Therapist I* is considered to be entry-level [16]. A between-subjects design is required as a participant’s experience in one condition can influence results in another condition if a within-subjects design is implemented. The assignment of a participant to either group will be randomly determined, subject to gender and experience balancing. This approach will result in four groups of 15 participants: trainee-control, trainee-ISO, therapist-control, and therapist-ISO. The experimental apparatus (including mockups) will be implemented for portability, and experiments may be conducted at several potential sites. We will approach students undergoing respiratory therapy training in Conestoga College. Experienced respiratory therapists will be recruited from hospitals and professional associations such as the Canadian Society for Respiratory Therapists and the Respiratory Therapy Society of Ontario. We may also approach practitioners via a booth setup in a conference focused in respiratory therapy. For both novice and experienced participants, inclusion criteria pertaining to prior experience with ventilator models from specific manufacturers will be applied. We will first distribute surveys to potential participants, wait for their responses, and select participants based on their familiarity with lung ventilator models from specific manufacturers. Figure 1 provides an overview of the planned timeline for recruiting clinical participants.

**Independent and dependent experimental variables.**

Dependent Variables

- Response accuracy: number of inaccurate mode or setting selections (in a scenario set of 10 scenarios)
- Average time for completion (taken across ten scenarios in one set)
- Responses to subjective ratings on interface evaluation questionnaire [17]

Independent Variables

- *Nomenclature type (between participants)*; Levels: Control versus ISO-Standard
- *Ventilator type (within participants)*; Levels: Familiar (PB-840) versus Unfamiliar (Engstrom)
- *Skill level (between participants)*; Levels: trainees versus experienced respiratory therapists

### Task 4: Experiment

The experiment is designed to simulate situations in which a clinician undergoes a transition from familiar to unfamiliar equipment, as such situations could give rise to clinician errors. Participants (both trainees and therapists) will be randomly placed in one of two groups: the control group or the ISO group. A summary of the experimental variables is presented in Textbox 1. Each group will experience a change of ventilators part way through the study, thereby modeling a transition from familiar to unfamiliar equipment in clinical settings. The control group will interact with existing nomenclature (similar to interactions in contemporary clinical settings) on each ventilator model. The ISO group will interact with the ISO DIS 19223 on each ventilator model. Therefore, the control group will first train on the PB-840 (with current manufacturer nomenclature), work on scenarios with the PB-840, and transition to the Engstrom (with current manufacturer nomenclature) after a short Engstrom training session. The ISO group will first train on the PB-840-ISO, work on scenarios with the PB-840-ISO, and transition to the Engstrom-ISO after a short Engstrom training session. These training sessions are intended to be very short. Thirty participants will be placed in each group (control and ISO), and each group will have an equal number of respiratory therapy students and registered respiratory therapists.

Experimental sessions may be videotaped, with the camera focused on the mockup screens only. All participants will be required to perform 10 scenarios on each ventilator model, which will include seven common scenarios that are routine in clinical settings and three emergency/nonroutine scenarios. Nonroutine scenarios or nonroutine events are events that would appear to be atypical to health care providers, may cause disruptions in the process of care delivery, and may result in cognitive deliberation in addition to what a routine event may demand; they also represent a class of events broader than *adverse events* [18]. Nonroutine events or scenarios can be helpful in capturing dysfunctional aspects of a clinical system or its perils [18], and could challenge cognitive processes and decision making [19]. Examples of routine scenarios are listed below; however, the scenarios in the final experiment will be different from the examples.

#### Example Scenario A

Set up the ventilator to the mode in which two pressure levels are set for spontaneous breathing, wherein the upper pressure should be set to 20 cmH2O, lower pressure set to 7 cmH2O, time at upper pressure should be 1.5 seconds, breathing rate set to 10, and percentage of oxygen by volume set to 28%. On the ventilator provided to you, change the settings to what would be appropriate for the patient.

#### Example Scenario B

Change the ventilator mode to the one in which pressure control inflations should be initiated at a rate of 10 while additional patient-trigger events would increase inflations of the selected type (ie, pressure control). Set inspiratory pressure to 15 cmH2O and baseline pressure at 5cmH2O above ambient pressure. The percentage of oxygen by volume should be 30%.

Participants will be instructed to respond to each scenario using their ventilator mockups, one at a time, and a time limit will be provided for each scenario. We will log start and end times for each scenario, which will be used to calculate response times for each scenario. When a participant does not respond accurately in a given scenario, the experimenter will probe the participant with questions. The inaccurate responses will also be recorded for analysis. Responses to these probes will be audio recorded, and they will be used to classify the error(s) based on Rasmussen’s error classification [20]. We may also use Reason’s classification [21] as suggested by the International Electrotechnical Commission [22]. At the end of each experimental session, participants will be required to complete a questionnaire to evaluate the interface. The evaluation questionnaire will be based on the one used by Salyer (67.7, page 1558) [17]. Salyer’s questionnaire will be modified to better suit the purpose of our evaluation protocol. Additionally, we will debrief the participants about the purpose of our study and about the possibility that vocabulary on future ventilator models may be standardized. The debriefing for the control group will include information about the nomenclature system in the ISO DIS 19223. In the debriefings, groups will be informed about how their ventilator interfaces differed from those in the other group.

**Figure 2:**
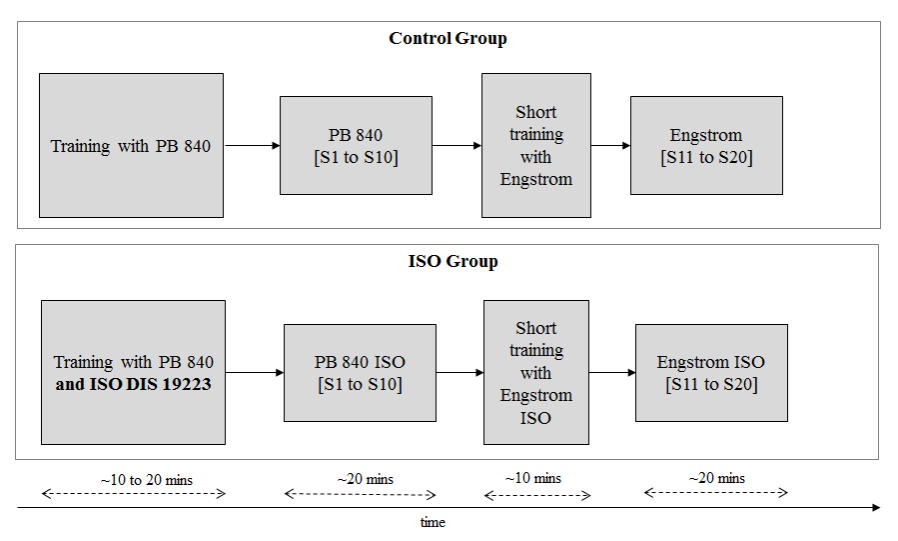
Timeline of an experimental session for the experiment. Each experimental session is expected to last approximately 80 minutes, including time for demographic surveys. PB-840: Puritan Bennett 840 ventilator; ISO: International Organization for Standardization; DIS: Draft International Standard; S: scenario set.

Figure 2 provides a summary of the planned timeline for an experimental session. Participants in the ISO group will be trained on the ISO DIS 19223. This training will familiarize participants with the terminology; however, participants will not be presented with a conversion table such as Table 1 that maps the ISO DIS 19223 to any manufacturer-specific terms. The scenario set (S1 to S10) on the PB-840 will be very similar to the scenario set (S11 to S20) on the Engstrom, to allow comparison of performance on the two types of ventilator models. Each scenario in the S1 to S10 set will have a corresponding scenario in the S11 to S20 set; however, the order of presenting these scenarios will be different.

### Task 5: Analysis and Reporting

Task 5 consists of statistical analyses and a qualitative analysis. The statistical analyses will mainly involve response times, accuracy, and subjective ratings, and will focus on performance differences between groups and ventilator types. We will run a mixed factorial analysis of variance to detect differences of statistical significance across groups and ventilator types. We will also apply statistical tests to detect any potential relationships between task performance and experience. Verbal responses to probe questions will be qualitatively analyzed [23]. The results of our proposed study will influence the ISO DIS 19223 standard for lung ventilators, and guide training programs and manuals provided by manufacturers. The insights will be useful to interface designers for lung ventilators. Additionally, frequently recurring errors associated with specific ISO DIS 19223 terms (if any) will be informative to the standards committee. We will also use the information from this study to provide guidance to respiratory therapists, colleges, and hospitals on how to manage the transition to ISO standard instrumentation.

## Results

The work reported here is in the proposal phase. Once the protocol is implemented, we will report the results in a follow-up paper.

## Discussion

### Executing the Protocol

In a number of domains in which time-critical tasks are performed with complex equipment, health care providers may be required to occasionally work with various manufacturers' models of equipment with which providers are unfamiliar. During such transitions, providers must cope with unfamiliar symbology, terminology, proprietary manufacturers' terms, audio tones, or patterns in interface navigation. Such transitions can be a cause for error, potentially leading to hazardous situations that result in environmental damage, patient morbidity, or mortality. To mitigate the risk of human error, efforts can be made to standardize terminology for instruction manuals, displays, and controls (eg, alarm signals of differing equipment used in critical care). Previous attempts to assess the usability of medical systems incorporating a specific terminology in the context of operation in realistic settings have been limited [7,24,25]. The protocol reported in our paper can be used to assess the role of standardization in mitigating the risk of human error in the use of devices that incorporate standardized terminology. This protocol provides templates for participant recruitment and experimental design. The protocol is also applicable in the evaluation of the vocabulary of terms proposed by the ISO DIS 19223 for lung ventilators. The proposed standardized vocabulary may have potential benefits for the usability of lung ventilators, and researchers around the world may be interested in performing similar studies to assess the potential benefits of the ISO DIS 19223. Medical equipment usability studies are required by many countries to obtain a license to sell the product in that country. The protocol reported here will assist researchers in recruiting participants and in designing the experiments to evaluate usability. Additionally, Table 1 provides a list of equivalent terms across those in the ISO DIS 19223 [6], the PB-840 [24], and the Engstrom [25]. Table 1 would be useful in designing studies involving these vocabularies. More information can be found in conversion tables provided by the Emergency Care Research Institute [26]; these tables provide comparisons across five terminologies, not including the ISO DIS 19223. The results from multiple studies informed by this protocol would help us understand potential benefits and difficulties associated with the use of the ISO DIS 19223.

### Limitations

We would like to indicate a few limitations in our protocol. Replication of this protocol for a planned experimental study in an industry or university laboratory would not be bound by the same limitations. First, there is a limit on the number of ventilator models that we can integrate into our study. The protocol reported in our paper includes only two ventilator models (PB-840 and the GE Engstrom). However, it is recommended to include more ventilator models, and we may include more ventilator(s) depending on availability of other ventilators and industry participation. Second, we will be using mockups, although it would be ideal to use the actual medical devices that have the ability to record data. Finally, nurses and clinicians other than respiratory therapists are not included in our protocol. Nurses may be regular operators of lung ventilators in some developing countries, and the inclusion of such nurses in an experimental protocol may be beneficial. A follow-up study can also be conducted to compare nurses with respiratory therapists.

## Abbreviations

ATIC: Architecture, Interface, Terminology, Information, Nursing, and Knowledge
BAP: baseline airway-pressure
CPAP: continuous positive airway pressure
DIS: Draft International Standard
GE: General Electric
ICU: intensive care unit
ISO: International Organization for Standardization
PB-840: Puritan Bennett 840 ventilator
PCV: Pressure Controlled Ventilation
PCV-VG: Pressure Controlled Ventilation - Volume Guaranteed
PEEP: positive end-expiratory pressure
SIMV: Synchronized Intermittent Mandatory Ventilation (in the ISO DIS 19223) or Synchronous Intermittent Mandatory Ventilation (in the PB-840)
SIMV-PC: Synchronized Intermittent Mandatory Ventilation - Pressure Controlled
SIMV-PCVG: Synchronized Intermittent Mandatory Ventilation - Pressure Controlled, Volume Guaranteed
SIMV-VC: Synchronized Intermittent Mandatory Ventilation - Volume Controlled
SNOMED CT: Systematized Nomenclature of Medicine - Clinical Terms
